# Clinical scores for outcomes of rhythm control or arrhythmia progression in patients with atrial fibrillation: a systematic review

**DOI:** 10.1007/s00392-017-1123-0

**Published:** 2017-05-30

**Authors:** Hai Deng, Ying Bai, Alena Shantsila, Laurent Fauchier, Tatjana S. Potpara, Gregory Y. H. Lip

**Affiliations:** 1 Institute of Cardiovascular Sciences, University of Birmingham, City Hospital, Birmingham, B18 7QH UK; 2 Guangdong Cardiovascular Institute, Guangdong General Hospital, Guangdong Academy of Medical Science, Guangzhou, China; 3 Beijing Tongren Hospital, Capital University, Beijing, China; 4 Service de Cardiologie, Centre Hospitalier Universitaire Trousseau, Tours, France; 5 School of Medicine, Belgrade University, Belgrade, Serbia; 6 Cardiology Clinic, Clinical Centre of Serbia, Belgrade, Serbia; 7 Aalborg Thrombosis Research Unit, Department of Clinical Medicine, Aalborg University, Aalborg, Denmark

**Keywords:** Atrial fibrillation, Rhythm control, Cardioversion, Catheter ablation

## Abstract

Patients with atrial fibrillation (AF) are commonly managed with rhythm control strategy, but the natural history of this common arrhythmia leads itself to progression from paroxysmal to persistent or permanent AF, and recurrences are evident despite rhythm control treatments using cardioversion or catheter ablation. Numerous clinical factors have been associated with outcomes of rhythm control or arrhythmia progression in patients with AF. The more common factors have been used to formulate risk stratification scores, to help predict the outcomes of rhythm control treatments or AF progression. This review article provides an overview on the published clinical risk scores related to outcomes of rhythm control strategy or AF progression.

## Introduction

Patients with atrial fibrillation (AF) are commonly managed with rhythm control strategy, but the natural history of this common arrhythmia leads itself to progression from paroxysmal to persistent or permanent AF, and recurrences despite rhythm control treatments using cardioversion or catheter ablation (CA). The latter has been shown to have superior efficacy in the short- or long-term comparison with antiarrhythmic drugs (AAD) even in patients with persistent AF [1, 2]. As an interventional procedure, CA therapy still has various issues including arrhythmia recurrence, procedural complications, and high expenditure which are important considerations when physicians have to make decisions whether CA is appropriate or not. Ultimately, a decision of atrioventricular junction ablation with permanent ventricular pacing may also be considered rather than AF ablation in highly symptomatic patients with high risk of AF recurrences [3].

The recurrence rate of a single CA procedure ranges from 30 to 50% [4]. Many clinical factors such as older age [5], non-paroxysmal AF [6], left atrial (LA) size [7], female sex [8], coronary artery disease (CAD) [4], hypertension (HTN) [9], diabetes mellitus (DM) [10], untreated obstructive sleep apnea (OSA) [4], metabolic syndrome (MetS) [11], body mass index (BMI) [12], chronic kidney disease (CKD) [13], heart failure (HF) [14, 15], early arrhythmia recurrence (ER) [16], and prior refractoriness to antiarrhythmic drugs [17] have been reported as predictor of recurrence after CA (see Table 1). Of the numerous clinical factors that have been associated with outcomes of rhythm control or arrhythmia progression in patients with AF, those which are more common have been used to formulate risk stratification scores, to help predict outcomes of rhythm control or arrhythmia progression. Several such scores have been published, as summarized in Table 2.

**Table 1:** Examples of risk factors for AF recurrence after catheter ablation

Risk factors	CA	CBA	References
LAD	√	√	Zhuang [7], Chao [57], Miyazaki [58], Liu [59]
LAD, PeAF, AF history	√		Miao [60]
Non-PAF	√		Konrad [6], Chang [61]
Epicardial adipose tissue thickness	√		Chao [62], Kim [63]
Early recurrence	√	√	Miyazaki [16], Evranos [64], Shim [65]
Obstructive sleep apnea	√		Naruse [66]
Inflammatory factors	√		Wu [67]
Duration of AF, gender	√		Zhang [68]
RA enlargement, ≥2 procedure times, AF duration	√		Zhao [69]
Uric acid, LAD, early recurrence		√	Canpolat [70]
Low BMI, PeAF	√		Fujino [71]
PR interval	√		Park [72]
Duration of AF, LAD, number of ineffective AAD	√		Takigawa [73]
Age, LAD, BMI, valvular heart disease, PR interval	√		Wu [74]
TGF-beta1		√	Canpolat [75]
Renal dysfunction		√	Li, 2014 [13], Neumann [76]
COPD	√		Gu [77]
Left ventricular systolic dysfunction	√		Anselmino [14]
Left ventricular diastolic dysfunction	√		Kumar [15]
Prior AAD failure, non-PAF, hypertension	√		Khaykin [17]
Hypertension	√		Wang [9]
Metabolic syndrome	√		Lin [11]
Diabetes mellitus	√		Anselmino [10]
Age	√		The [5]
Female	√		Zylla, 2016 [8]

*AAD* antiarrhythmic drug, *AF*, atrial fibrillation, *BMI* body mass index, *CA* catheter ablation, *CBA* cryoballoon ablation, *COPD* chronic obstruct pulmonary disease, *LAD* left atrial diameter, *non-PAF* non-paroxysmal AF, *PeAF* persistent AF

**Table 2:** Studies for predictive scores related to outcomes of rhythm control or arrhythmia progression in patients with atrial fibrillation

First author, year	Scores (points)	Enrolled patients (n)	pAF (%)	CA protocol	AFLAT-Free (%)	Procedure times	FU (months)	C Index**/**AUC	Predictive value	CHADS_2_/CHA_2_DS_2_–VASc compared
Tang [21], 2012	HATCH (0–7)	488	100	CPVI	63.93	Single	27.4 ± 17.7	Not measured	No	Not compared
Maciej [23], 2013	ALARMEc (0–5)	213	47	Stepwise*	90	Repeated	24	0.657 vs. 0.533/0.519	Yes	Worse
Ugur [25] 2013	BASE-AF_2_ (0–6)	236	79.6	Cryoablation	74.5	Single	20	0.94 (score ≥3)	Yes	Not compared
Letsas [31] 2014	CHADS_2_ (0–6)/CHA_2_DS_2_–VASc (0–9)	126	100	CPVI	70.6	Single	16	0.644/0.627 (score ≥2)	Yes	–
Kornej [26], 2015	APPLE (0–5)	261	48	Stepwise*	38.3	Single	≥12	0.634 vs. 0.538/0.542	Yes	Worse
Roger [28], 2016	CAAP-AF (0–13)	937	31.6	Stepwise*	79.1	Repeated	21.6 ± 1.6a	0.650	Yes	Not compared
Mujovic [29], 2017	MB-LATER (0–6)	133	69.2	Stepwise*	85	Repeated	29 ± 10.1	0.782 vs. 0.552/0.519	Yes	Worse

*AFLAT* atrial flutter or atrial tachycardia, *AUC* area under curve, *CPVI* circumferential pulmonary vein isolation, *FU* follow-up, *pAF* paroxysmal atrial fibrillation

* Stepwise, necessary additional linear lesion, or complex fractionated atrial electrogram-guided ablation after CPVI

The objective of this review article is to provide an overview on the published clinical risk scores related to outcomes of rhythm control strategy or arrhythmia progression.

### Search strategy

Comprehensive literature search was performed using MEDLINE for studies reporting on the predictive scores of AF recurrence after CA or AF progression. Search terms included “atrial fibrillation”, “ablation”, “recurrence”, “outcome”, “progression”, and “score”. The articles retrieved by the search were selected by title and abstract screening. Nine relevant clinical scoring systems have been reported (Table 2). We summarized prediction, with c-indexes or area under the curve (AUC), or reclassification or discrimination indexes, where reported. As most clinical scores only had one associated paper related to rhythm control or arrhythmia progression, no meta-analysis was performed, given the heterogeneity of the patient populations studied.

### The HATCH score

The HATCH scoring system was first developed to predict the clinical progression of paroxysmal to persistent AF [18]. In the original description, 1219 patients from the Euro Heart Survey on AF were included and were observed for more than 1 year. Different clinical variables were studied to evaluate the predictive value on AF progression, and heart failure (H), older age (A), previous transient ischemic attack (TIA) or stroke (T), chronic obstructive pulmonary disease (COPD,C), and hypertension (H) were identified as independent predictors of AF progression.

Hypertension, age ≥75 years, and COPD each were assigned 1 point, while TIA or stroke, and heart failure were scored 2 points, with the HATCH score ranging from 0 to 7 points. In the original study, nearly 50% of patients with a HATCH score of >5 progressed to persistent AF, but only 6% of those with a score of 0 experienced AF progression. A subsequent study of AF patients who were awaiting CA found the HATCH score to be a poor predictor of AF progression [19]. Similar observations were seen in the Belgrade AF study, which showed that the HATCH score had only very modest predictive value (c statistic, 0.6) for the arrhythmia progression in a cohort of lone AF patients over a 12-year follow-up period [20].

Whether the HATCH score could be used to predict the outcome after CA of AF was studied by Tang et al. [21] in 488 patients with paroxysmal AF undergoing CA. After 27.4 ± 17.7-month follow-up, 69.93% of patients were free of late AF recurrence, but the HATCH score was not an independent predictor of recurrence on multivariable analysis. Thus, the HATCH could not reliably predict the outcome after CA.

The predictive value of the HATCH score was further explored recently. Suenari et al. [22] tested the score in a 670,804 patients’ cohort to investigate its predictive value of new-onset AF. In this cohort, patients were 20 years older, than in derivation cohort, without AF history. During a follow-up of 9.0 ± 2.2 years, the AF incidence increased from 0.8 per 1000 patient-years for patients with a HATCH score of 0–57.3 per 1000 patient-years for those with 7. After adjustment for gender and comorbidities, the hazard ratio of each increment of the HATCH score in predicting new-onset AF was 2.059 (CI 2.027–2.093, *P* < 0.001). The result showed that the HATCH score was useful in estimation and stratification of new-onset AF.

#### The ALARMEc score

The ALARMEc score [23] was first reported as a scoring system to predict the outcome of AF CA in a study comparing the ALARMEc score with the CHADS_2_ and CHA_2_DS_2_–VASc scores for stroke risk stratification. The ALARMEc score included five variables, as follows: AF type (A), Left Atrial size [normalized left atrial area (NLA) ≥10.25], Renal insufficiency (eGRF <68 ml/min), Metabolic syndrome and cardiomyopathy (c) with each variable scoring 1 point, and the score values ranging from 0 to 5 points. The ALARMEc score was tested in only 213 patients with paroxysmal AF or non-paroxysmal AF who underwent repeated CA. After a follow-up of up to 60 months, only the ALARMEc score (AUC 0.657, *P* < 0.0001) but not CHADS_2_ (AUC 0.533, *P* = 0.413) or CHA_2_DS_2_–VASc (AUC 0.519, *P* = 0.641) score predicted outcomes after CA.

Another study [24] included 702 patients with AF and analyzed four of five risk factors in a modified (ARLAMEc) score. In this study, MetS and impaired renal function were independent predictors of AF postablation outcome, but NLA and AF type were non-predictive. For the outcome of repeated CA, MetS was not predictive for late recurrences, but impaired renal function remained a significant predictive factor. Result of this study had some difference from the previous one.

### BASE-AF2 score

The BASE-AF2 score system [25] comprises six clinical variables, as follows: Body mass index (BMI) >28 kg/m^2^ (B), atrial dilatation >40 mm (A), current smoking (S), early recurrence (E), duration of AF >6 years (A), and non-paroxysmal AF type (F). Each variable scores 1 point, with the score range from 0 to 6 points.

This score was tested in a set of 236 patients with paroxysmal AF who underwent cryoablation, and those with AF recurrence had higher BASE-AF2 score values, with a score of ≥3 points being an independent predictor of AF recurrence. This score was only tested in patients with AF undergoing cryoablation and its value in other CA modalities merits further study.

### The APPLE score

The APPLE score [26] was derived from a cohort of AF patients from Germany and tested in a validation cohort from the US. This scoring system comprised of five variables, as follows: Age >65 years (A), persistent AF (P), impaired eGFR (<60 ml/min/1.73 m2) (P), LA diameter ≥43 mm (L), and EF <50% (E). Each variable scored 1 point with the score ranging from 0 to 5 points.

In the derivation cohort, logistic regression analyses showed that the APPLE, CHADS_2,_ and CHA_2_DS_2_–VASc scores were significant predictors of AF recurrence between 3 and 12 months. Based on ROC curve analysis, the APPLE score had a better predictive value compared with CHADS_2_ and CHA_2_DS_2_–VASc score (*c* index 0.634 vs. 0.538 and 0.542, respectively, both *P* < 0.001). Validation study [27] of the APPLE score also carried on a cohort (*n* = 379) under repeated CA. Compared with CHADS2 and CHA_2_DS_2_–VASc score, the APPLE score also had better predictive value of AF recurrence after repeated CA (AUC 0.617 vs. 0.577 and 0.590, respectively, both *P* < 0.001). In the latest validation study, the risk (OR) of AF recurrence was 2.9, 3.0, and 6.0 for patients with APPLE score 1, 2, and ≥3, respectively, when compared to an APPLE score of 0 (all *P* < 0.01).

### The CAAP-AF score

The CAAP-AF score [28] was initially described in a derivation cohort of 1125 AF patients and tested in a validation cohort of 937 AF patients who underwent first CA at the same centre. AF type included paroxysmal AF and non-paroxysmal AF. The score consisted of the following variables: CAD (C), LA diameter (A), age (A), persistent or long-standing AF (P), number of antiarrhythmic drugs failed (A), and female sex (F), which were independent risk factors of AF recurrence in the derivation cohort on multivariable analysis. Accordingly, the C, F, and P criteria were awarded 1, 1, and 2 points, respectively. The score was scored 0–4 based on the different LA diameters of <4.0, 4.0 to <4.5, 4.5 to <5.0, 5.0 to <5.5, and ≥5.5 cm. The age criterion was given different points of 0–3 for ages <50, 50 to <60, 60 to <70, and ≥70 years. If patient had 1 or 2 antiarrhythmic drug failures, it scored 1 point. When the number was over 2, it scored two points.

Thus, the CAAP-AF score system comprises six risk factors and the score ranges from 0 to 13 points. In the validation cohort, percentage of AF-free patients was identical to that seen in the derivation cohort (statistic C 0.650 vs. 0.691). Of note, the CAAP-AF score was based on a single centre experience and AF recurrence was detected only by 24 h or 7-day Holter but not by loop recorder implantation.

### The MB-LATER score

The recently published MB-LATER score was recently proposed [29] to predict very late (>12 months) recurrence of AF (VLRAF) after CA. In this score, five clinical factors are considered: male sex, bundle branch block, left atrial size ≥47 mm, type of AF (paroxysmal, persistent, or long-standing persistent), and early recurrent AF. The MB-LATER was derived from a small retrospective cohort and compared against other clinical scores (APPLE, ALARMEc, BASE-AF2, CHADS2, CHA_2_DS_2_–VASc, or HATCH). When compared to these scores, the MB-LATER demonstrated better predictive value (AUC 0.782 vs. 0.716, 0.671, 0.648, 0.522, 0.519, or 0.583) and improved identification of patients with subsequent VLRAF using decision curve analysis (DCA).

### The CHADS_2_, CHA_2_DS_2_–VASc, and R_2_CHADS_2_ scores

The CHADS_2_ (congestive heart failure, hypertension, Age ≥75, diabetes mellitus, and stroke/transient ischemic attack), CHA_2_DS_2_–VASc (congestive heart failure, hypertension, age ≥75 years, diabetes mellitus, stroke/transient ischemic attack, vascular disease, age 65–74 years, and female sex), and R_2_CHADS_2_ (renal dysfunction, congestive heart failure, hypertension, age ≥75 years, diabetes mellitus, and stroke/transient ischemic attack) scores are risk scores for predicting stroke and thromboembolism [30]. Given that the risk factor components of these scores are common cardiovascular risk factors, it is no surprise that they can be [29] related to outcomes of rhythm control or arrhythmia progression.

For example, Letsas et al. reported that on univariate analysis, both CHADS_2_ and CHA_2_DS_2_–VASc scores were associated with AF recurrence in patients with paroxysmal AF undergoing a single CA procedure. A score of ≥2 for both CHADS_2_ (AUC 0.644) and CHA_2_DS_2_–VASc (AUC 0.627) scores had the highest predictive value for AF recurrence [31]. Another study [32], which included patients with paroxysmal AF and persistent AF, reported that both CHADS_2_ (HR 1.19, *P* < 0.001) and CHA_2_DS_2_–VASc (HR 1.15, *P* < 0.0001) scores were good in stratifying patients for 5-year outcomes after AF ablation, with the CHA_2_DS_2_-VASc (HR 1.13, *P* = 0.001) score being superior to the CHADS_2_ score for predicting AF recurrence.

Kornej et al. [33] reported that AF type, LA diameter, and early recurrence (ER) were significant predictors of long-term recurrence post AF ablation, and not the CHADS_2_, CHA_2_DS_2_–VASc, and R_2_CHADS_2_ scores. Reports on the predictive value of these stroke risk scores on rhythm control outcomes do not appear to have consistent results.

### A critique of the published scores

AF recurrence after CA was defined as AF/AT/AFL episode lasting 30 s with or without symptom recorded over 3 months after the procedure in the derivation or validation research of all the clinical scores.

Scoring systems described above used different predictive clinical factors in combination (see Table 3). Some of these factors like BMI and MetS had conflict results [11, 12]. One meta-analysis found that recurrence within 30 days, LA diameter of >50 mm, and valvular AF were the most powerful predictors of CA failure [34].

**Table 3:** **Scoring systems and risk factors included**

Risk score	Points	Age	gender	AF type	AF history	LAD	ER	HTN	CAD	HF	DM	CKD	Smoking	TIA/stroke	BMI	COPD	AAD F	MetS	CMD	Vsc D	BBB
HATCH	0–5	√						√		√				√		√					
ALARMEc	0–5			√		√						√						√	√		
BASE-AF_2_	0–6			√	√	√	√						√		√						
CHADS_2_	0–6	√						√		√	√			√							
R_2_CHADS_2_	0–8	√						√		√	√	√		√							
CHA_2_DS_2_–VASC	0–9	√	√					√		√	√			√						√	
APPLE	0–5	√		√		√				√		√									
CAAP-AF	0–13	√	√	√		√			√								√				
MB-LATER	0–6		√	√		√	√														√

*AAD F* prior antiarrhythmic drug failure, *BMI* body mass index, *CAD* coronary heart disease, *CKD* chronic kidney disease, *CMD* cardiomyopathy disease, *COPD* chronic obstruct pulmonary disease, *DM* diabetes mellitus, *ER* early reference, *HTN* hypertension, *LAD* left atrial diameter, *MetS* metabolic syndrome, *TIA* transient ischemic attack, *Vsc D* vascular disease, *BBB* bundle branch block

From the clinical perspective, AF progression could be defined as development of persistent or long-standing AF in patients with paroxysmal AF. In the derivation study of HATCH score, five clinical factors were identified as independent predictors of AF progression (see Table 3). Although these factors have previously been reported separately (see Table 1), the intrinsic mechanistic link and the development of the substrate for AF or its progression requires further study. The predictive value in AF progression of the HATCH score is still controversial, but result of recent validation study on new-onset AF demonstrated good predictive ability. Clinical applicability of the HATCH score needs much more evidence to support.

Five scores derived from different cohorts, that is, the ALARMEc, BASE-AF2, APPLE, CAAP-AF, and MB-LATER, included the LA size as one of the predictive variables. Nonetheless, the size of LA was differently defined in different scores. For example, LA size was defined as >43 mm in APPLE score, >40 mm in BASE-AF2 score, >47 mm in MB-LATER, and >50 mm in ALARMEc score, and stratified into five categories of <40, 40 to <45, 45 to <50, 50 to <55, and ≥55 mm in the CAAP-AF score. Given that they were not initially developed to predict the rhythm outcome following CA, the CHADS_2_, CHA_2_DS_2_–VASc, R_2_CHADS_2,_ and HATCH scores did not include LA size as one of their component risk factors.

LA enlargement is involved in mechanism of AF formation and progression. Atrial fibrosis may be an important feature for AF perpetuation, and it may be evaluated using cardiac imaging [35] or biomarkers [36–38]. Interestingly, fibrosis may be found in AF patients with no LA enlargement. Whether these parameters may improve the predictive value of scores aiming to identify the risk of arrhythmia progression should be evaluated in the future. Some of the included risk factors could relate to pathophysiological changes in the LA. For example, patients with HTN were found to have increasing size of scar and low-voltage area in the LA when mapping during the CA procedure [9]. MetS may play an important role in the atrial electrical activity by promoting the atrial conduction disturbances and dispersion of refractoriness between the right and left atrium [39]. In addition, obesity [40] is associated with a shortened effective refractory period in the pulmonary veins.

AF subtype was another variable included in five scoring systems (see Table 3). Electrical changes promoting arrhythmia perpetuation are induced by the presence of AF itself, which has also been called “AF begets AF” several years ago. From the onset of AF, the LA undergoes gradual electrical and structural remodeling which ultimately forms a substrate capable to maintain AF. Non-paroxysmal AF has been associated with lower AF termination rates and worse outcome after CA compared to paroxysmal AF [6]. The predictive value of AF duration for postablation recurrence of non-paroxysmal AF has been reported widely [6, 17, 41].

Early AF recurrence was only included in the BASE-AF2 and MB-LATER score. As we know, early recurrence is observed only after CA, and while of limited use in pre-ablation decision, it might be useful to predict rhythm outcome following repeated ablation procedures, as a reconnection of the PV–LA electrical conduction is considered to be the main mechanism of ER [16] as well as recurrence [42]. Thus, LA size, AF type, and ER, which may directly contribute to the AF substrate, should be considered as most important parameters included within the five main scoring systems.

Other risk factors were shared in several scores. Age was risk factor of the HATCH, CHADS_2_, R_2_CHA_2_DS_2_, CHA_2_DS_2_–VASc, APPLE, and CAAP-AF scores. Heart failure (HF) was shared in the HATCH, CHADS_2_, R_2_CHA_2_DS_2_, CHA_2_DS_2_–VASc, and CAAP-AF score. These two factors were previously reported elsewhere. Incidence of HF is increased in AF patients which encourage a rhythm control strategy [43]. Vice versa, patients with HF progress to AF more easier than those without and evidence showed that risk profile is shared by HF and AF [44].

Of the five mentioned scores, the ALARMEc, BASE-AF_2_, APPLE, CAAP-AF, and MB-LATER scores each were tested in only one study, and these studies included patients with different AF types. Only the BASE-AF_2_ score used the cryoablation technique which could have had some influence on the outcome of non-paroxysmal AF patients. Indeed, cryoablation just performs a circumferential pulmonary vein isolation (CPVI) and the result in non-paroxysmal AF ablation may potentially be suboptimal with cryoablation [25], although the optimal strategy for ablation in non-paroxysmal AF still needs to be established [45]. Compared to the MB-LATER score, the BASE-AF2 score also had moderate predictive value (AUC 0.648) for VLRAF [25].

The other four studies used radio frequency CA and a stepwise protocol, especially with non-paroxysmal AF, where patients would sometime need to have linear lesions (LL), mitral/tricuspid isthmus ablation, superior vena cava isolation, or complex fractionated atrial electrogram ablation (CFAE) when AF is continuous after the CPVI [23, 26, 28]. There were minor differences among the approaches used in these four studies. Patients from the CAAP-AF cohort underwent CPVI and roof linear lesions in both paroxysmal or non-paroxysmal AF, and coronary sinus ablation was performed. In the derivation cohort of the APPLE score, electrical cardioversion was used initially if AF presented at the beginning of the CA procedure. However, in the validation cohort, cardioversion was used when AF was continuous after CPVI, roof linear lesion, mitral linear lesion, base posterior wall lesion, or CFAE, although the efficacy of additional linear lesion and CFAE on sinus rhythm maintaining after CA has not been firmly established [46, 47]. None of these studies provided much detail on ablation parameters, complication, and AF termination of the procedure, which might influence the acute procedural rate of AF. As far as we known, new techniques are evolving within CA such as contact force catheter [48] and second-generation cryoballoon [49], new mapping systems (e.g., focal impulse and rotor or high dominant frequency mapping) [50, 51], and new ablation technique (e.g., hybrid or epicardial ablation) [52] have influenced the efficiency and safety of AF ablation. Hence, the predictive value of these clinical scores would need to be validated in cohorts undergoing ablation with newer ablation catheters or techniques.

The follow-up period of five derivation studies has some differences (see Table 2). Patients in these studies were followed up at least 12 months. Arrhythmia symptom with 12-lead ECG evidence and continuous Holter ECG monitor were used to detect AF recurrence. Patients accepted 7-day Holter for every 3 months in studies on the ALARMEc and APPLE scores, and then once a year in the study on the ALARMEc score. Of the validation cohort of the CAAP-AF score, patients accepted 24–48 h Holter every 3–12 months but switched to 7–14-days Holter after 2006. Patients of the BASE-AF2 cohort were only accepted with 24 h Holter every 3 months. In the MB-LATER cohort, patients underwent 12-lead ECG and 24 h Holter at discharge, 1, 3, and 6 months after procedure, and then every 6 months thereafter. If patients complained with symptoms suggestive of arrhythmia recurrence, more extensive arrhythmia monitoring would be performed. Asymptomatic AF sometimes occurred much more frequently than symptomatic AF [53]. None of these studies use implanted recorder to detect the arrhythmia recurrence which makes their estimation of AF recurrent rate suboptimal. For expense or non-invasive reason, wearable instrument which was usually used to avoid unnecessary ICD implantation might take as the substitution [54]. Testing studies on these scores with much precise recurrent AF rate may help to improve overcome this limitation. Some evidence has shown that the two ablation techniques with cryoablation and radiofrequency had similar efficacy [49] in patients with paroxysmal AF, but evidence in persistent AF is scarce.

The CHADS_2_, CHA_2_DS_2_–VASc, and R_2_CHADS_2_ scores, which are not rhythm-related risk scores, were tested in different cohorts with conflicting results. The HATCH score had no significant value on predicting the recurrence after CA [21]. Three *clinical* scores determined before the CA procedure could be used to predict AF recurrence post CA. In the studies describing the ALARMEc and CAAP-AF scores [23, 28], the scores’ predictive value for repeated CA outcome was also evaluated. Validation tests of the ALARMEc score were contradictory and the CAAP-AF score was not validated by any other cohort. For now, the APPLE score has been validated in at least three cohorts (AUC 0.634, 0.617, and 0.716, all *P* < 0.05) including the derivation cohort of MB-LATER score. Risk factors of the APPLE score are easily acquired clinical indices, which makes this score a good predictor of AF recurrence post CA.

For early recurrence based on clinical factors after the CA procedure, the BASE-AF2 and MB-LATER scores [25, 29] could be used to predict AF recurrence post CA. The predictive value of BASE-AF2 is perhaps more limited given the derivation study design. The MB-LATER score was newly derived and validated for the predictive value of VLAFR and compared to the other six scores except for the CAAP-AF score. The MB-LATER score was shown to have better predictive value for VLAFR than other scores in a small prospective cohort (*n* = 133) study. Although there is only one report for now, the MB-LATER score appeared to be a good tool to predict VLAFR, but the value for AF recurrence after CA needs to be further validated. In the validation studies of the APPLE and the MB-LATER score, different points or cut-off analyses were carried out. Overall, the predictive value of all these scores still requires more validation studies to help decision-making on AF recurrence ablation or postablation outcomes.

### Limitations

The majority of the derivation studies used to develop these scoring systems had observational retrospective designs and some scores lacked external validation cohorts. In addition, patients who undergo cryoablation usually have less risk factors. Based on our review, all scores were derived from different cohorts, which made components of them rather different. Our purpose was to report every clinical score that had been derived and try to compare their reported clinical predictive value(s) in relation to CA method(s). While we fully recognize that cryoablation is one type of ablation method/technology, but a recent report from the Fire and Ice trial [ref] showed it had similar outcomes to PAF patients undergoing RF ablation. Persistent AF may need additional ablation approaches, such as linear lesion (LL), but meta-analysis does not suggest that LL following pulmonary vein isolation (PVI) provides additional benefit for sinus rhythm maintenance. Given the possible heterogeneity of reported cohorts and also that our focus was not on ablation technique, our review does not focus on outcomes in relation to comparison of ablation methods. Finally, the predictive value of the scores on AF progression and recurrence requires to be confirmed in future studies. Large cohorts should be used to test all these scores to confirm their clinical applicability. Case studies from different centres often not large, multicentre clinical trial data or different centre combining the data might be of help like the derivation of the TIMI-AF score [55] and the AF-CVS score [56]. Until large prospective cohorts exist, we should regard application of these scores as hypothesis generating, but using these scores may provide some insights on who may (or may not) do well following ablation.

## Conclusion

Several predictive scores for rhythm outcome of AF recurrence postCA have been developed and tested, but evidence of their predictive value still requires further evaluation. Many risk factor components of these scores have been reported as independent predictors of CA outcome, whether directly or indirectly contributing to AF substrate formation. For now, the risk scores for recurrences following CA have limited validation.
